# Comparative efficacy of chitosan and calcium carbonate-based scaffolds for bone regeneration in preclinical in vivo models: a systematic review

**DOI:** 10.1007/s44445-026-00187-3

**Published:** 2026-06-01

**Authors:** Wilman Rante Marampa, Renny Febrida, Nina Djustiana

**Affiliations:** 1 Master of Dental Science, Padjadjaran University, Bandung, Indonesia; 2 Department of Dental Materials Science and Technology, Padjadjaran University, Bandung, Indonesia; 3 Oral Biomaterial Study Center, Padjadjaran University, Bandung, Indonesia

**Keywords:** Chitosan, Calcium carbonate, Scaffolds, Bone tissue engineering, Bone regeneration

## Abstract

**Purpose:**

To investigate the efficacy of chitosan and calcium carbonate (CaCO₃)-based scaffolds in promoting bone regeneration in preclinical in vivo models, focusing on bone volume, mineral density, histological outcomes, and biomechanical properties.

**Methods:**

This systematic review adheres to PRISMA guidelines. A comprehensive search was conducted across PubMed, Web of Science, Scopus, and Cochrane Library for studies published from March 2015 to March 2025 using keywords such as “chitosan,” “calcium carbonate,” “bone regeneration,” and “scaffolds.” Eligible studies involved preclinical in vivo research on bone defect repair with chitosan or calcium carbonate scaffolds. Studies were included if they compared these scaffolds with other bone regeneration materials or no intervention. The methodological quality of the studies was assessed using the SYRCLE Risk of Bias tool.

**Results:**

Thirty preclinical in vivo studies involving 850 animals were included. Chitosan scaffolds showed new bone formation ranging from 40% to 85%, while calcium carbonate scaffolds showed regeneration rates between 50% and 70%. Significant improvements in bone mineral density (BMD), bone volume-to-total volume ratio (BV/TV), and trabecular thickness were observed in scaffold-treated groups. Histological findings indicated increased osteoblast activity, mineralization, and vascularization, especially in chitosan combined with other materials. No significant biomechanical differences were found between the scaffolds, but composite materials showed improved mechanical properties (*p* < 0.05).

**Conclusions:**

Both chitosan and calcium carbonate–based scaffolds show promising potential in preclinical models, with chitosan reporting 40–85% new bone formation (≈19 studies) and CaCO₃ reporting 50–70% (≈11 studies). However, heterogeneity in study design and reporting quality warrants cautious interpretation. This variability also underscores the need for standardized testing and further research to isolate the independent effects of scaffold composition.

**Graphical Abstract:**

**Figure:**
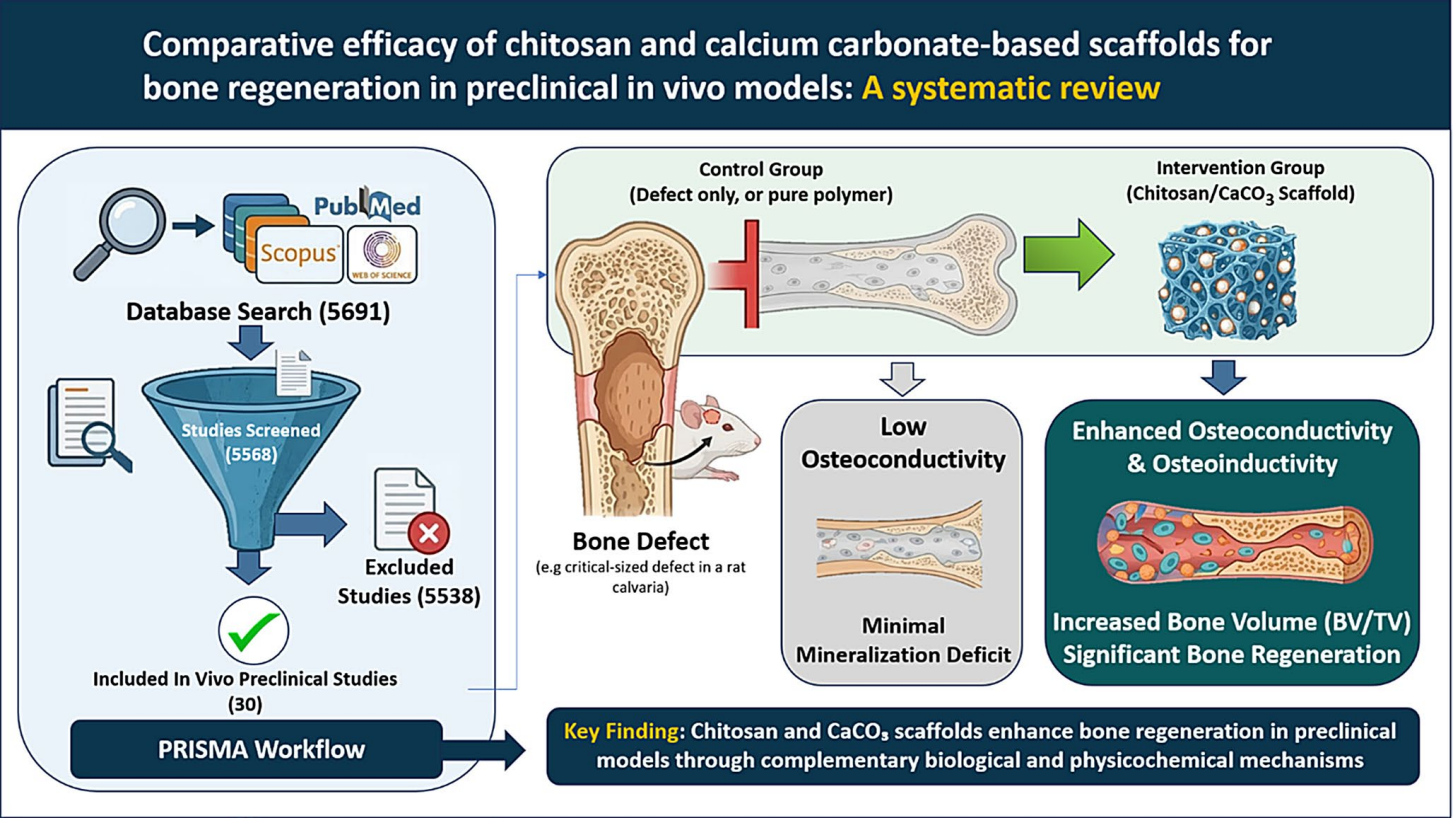
Overview of a PRISMA-based systematic review evaluating chitosan (CS) and calcium carbonate (CaCO₃) scaffolds for bone regeneration in preclinical animal models. Both materials enhance osteogenesis and bone defect repair through complementary biological (CS) and physicochemical (CaCO₃) mechanisms.

## Introduction

Bone defects, especially those with significant tissue loss, are a major challenge in oral and maxillofacial surgery. These defects often happen due to trauma, disease, or surgical procedures, leading to non-union and requiring intervention to restore both structure and function (Arash and Hosseinpour 2020; Li et al. 2021; Michael and Hagenmaier 2002). Traditional treatment options, such as autografts, allografts, and synthetic implants, have their limitations, including issues like donor-site morbidity, the risk of disease transmission, immune reactions, and high costs (Kremmyda 2022; Farré-Guasch et al. 2013). In more complicated cases, like intra-articular fractures or severe trauma, current reconstruction methods often do not result in bone regeneration that matches the quality of native bone in terms of both biomechanics and histology (Devireddy et al. 2015; Jiang et al. 2024). These challenges highlight the need for better bone tissue engineering approaches that can promote regeneration with fewer risks and complications.

Tissue engineering has become a promising approach to addressing these challenges, primarily through the development of biodegradable scaffolds that facilitate bone regeneration. These strategies provide alternatives to autografts and allografts by utilising synthetic scaffolds to stimulate tissue growth. A scaffold acts as a filler in the defect area, helping to promote bone healing (Agnes et al. 2023; Levengood and Zhang 2014). Among various materials, chitosan (CS) and calcium carbonate (CaCO₃) have gained attention because they are biocompatible, osteoconductive, and can mimic the mineral makeup of bone (Terzioğlu 2021). Chitosan-based scaffolds have shown great potential. They support osteoblast attachment, help cells multiply, and promote the formation of a mineralised matrix. It can be shaped into different 3D structures with adjustable porosity and mechanical strength. Recently, advancements have also been made in creating composite scaffolds that combine chitosan with ceramics or other polymers to enhance both mechanical strength and biological performance (Agnes et al. 2023; Levengood and Zhang 2014; Terzioğlu 2021).

Despite the growing body of literature on chitosan and calcium carbonate (CaCO₃)-based scaffolds, most in vivo studies include additional bioactive agents, such as growth factors, stem cells, or pharmaceuticals. This makes it difficult to evaluate the scaffolds’ independent regenerative potential. Previous studies have generally focused on bone tissue engineering materials or cell-based therapies, but few have isolated chitosan and calcium carbonate (CaCO₃) as the primary biomaterials in animal models. Furthermore, while some studies report excellent osteogenic outcomes with chitosan-CaCO₃ scaffolds, others show more modest or inconsistent results. This variability may arise from differences in animal species, defect models, scaffold fabrication methods, and evaluation metrics, highlighting the need for systematic synthesis to clarify the regenerative potential of these biomaterials. That is why this review will look at how effective these materials are and assess results using measurable factors, like the ratio of bone volume to total volume (BV/TV), bone mineral density (BMD), tissue structure findings, and strength of the bone.

Therefore, this systematic review aims to comparatively evaluate the efficacy of chitosan- and calcium carbonate (CaCO₃)-based scaffolds in preclinical in vivo bone defect models. Specifically, this review seeks to (Arash and Hosseinpour 2020) synthesize quantitative evidence on bone regeneration outcomes (e.g., BV/TV, BMD, histological bone formation, and biomechanical parameters) (Li et al. 2021), analyze how scaffold composition and biological augmentation influence osteogenic performance, and (Michael and Hagenmaier 2002) assess the translational relevance of different defect models and load-bearing conditions. By applying a structured comparative framework, this review aims to clarify the intrinsic regenerative potential of chitosan and CaCO₃ scaffolds and identify conditions under which their performance is most clinically meaningful.

## Materials and methods

### Data collection

The data collection for this systematic review complies with the Preferred Reporting Items for Systematic Reviews and Meta-Analyses (PRISMA) guidelines to guarantee transparency, reproducibility, and methodological integrity. The reviewers evaluated studies identified through extensive electronic searches of prominent bibliographic databases, including PubMed/MEDLINE, Web of Science, Scopus and Cochrane Library from 1 March 2015 to 1 March 2025 for publications. The search strategy utilized combinations of pertinent keywords, including “chitosan”, “scaffold”, “animals”, and “bone regenerate”. along with synonyms and variations to encompass the entirety of eligible studies. The detailed search terms are given in Appendix 1.

### Eligibility criteria

The studies included were in vivo preclinical studies involving experimental animals (such as mice, rabbits, and others) that clearly defined bone defect models and utilized chitosan-based, calcium carbonate (CaCO₃)-based, or a combination of both scaffolds. Research was required to compare these scaffolds with other bone regeneration scaffolds (such as hydroxyapatite, collagen, etc.) or with a negative control (no scaffold). Only studies that reported the efficacy of scaffolds in bone regeneration were included, evaluating outcomes such as the rate of new bone tissue formation, histopathological results (such as bone matrix deposition and mineralization), mechanical evaluation of the formed bone, and improvements in bone tissue function or integrity. Studies must have clearly defined experimental designs and used systematic methods. Only studies published in English were considered. Exclusion criteria encompassed in vitro or cell-based studies, studies not using in vivo animal models, and studies that did not involve chitosan or CaCO₃ scaffolds. Articles that focused solely on secondary outcomes without reporting bone regeneration, review articles, editorials, case reports, conference abstracts, or articles published more than 10 years ago were excluded. Studies without full-text access, those without adequate experimental designs (such as those without a control group), or those lacking clear bone regeneration results were also excluded.

### Study selection

Keywords were employed to identify potential studies for inclusion in the search. Following the elimination of duplicates via Rayyan.ai, reviewers evaluated all the retrieved studies based on their titles and abstracts. All phases of screening were conducted utilizing the Rayyan.ai program. Thorough evaluations were conducted to determine if a full-text study could be conducted using the previously specified eligibility criteria. Reviewers consistently established a consensus to address any discrepancies or ambiguities that arose during the selection process. Any discrepancies were resolved through discussion until consensus was reached.

### Data extraction

Each study included in this review was characterized in a separate table, followed by the data extraction presented in another table. The characterization for each study in this review included: 1) Author(s) and publication year; 2) Animal species, strain, sex, age, weight, and the 3) total sample of animals; 4) Bone defect model, including defect location, size, and healing period. 5) Duration of the study. The extracted data from each study were presented in the following order: 1) Type of scaffold used, including composition, fabrication method, and physicochemical characteristics of the chitosan and/or calcium carbonate (CaCO₃)-based scaffold; 2) Comparator details, including control groups such as untreated defects, empty scaffolds, or alternative biomaterials; 3) Outcome measures, which included evaluations of bone regeneration using radiological (e.g., micro-CT, BMD, BV/TV), histological (e.g., matrix mineralization, osteoid formation), and biomechanical (e.g., strength, stiffness) parameters, when available.

### Quality assessment

The SYRCLE Risk of Bias (RoB) tool was used to evaluate the methodological quality and risk of bias in the included studies. This tool assesses various types of bias, including selection, performance, detection, attrition, reporting, and other potential sources not explicitly covered. The evaluation was conducted based on 10 items, where each item was answered with “Yes,” “No,” or “Unclear” (see Appendix 2). A study was considered to have a low risk of bias (RoB) if all sub-items were answered with “Yes,” a high RoB if at least one sub-item was answered with “No,” and an unclear RoB if the answers were mixed or uncertain. For each study, the overall risk of bias was classified as low if all items were rated as “Yes,” high if any item was rated as “No,” and “Unclear” if the responses were inconsistent.

## Results

### Study selection

A search across four electronic databases resulted in a total of 5,691 articles. After screening titles and abstracts, 82 articles were selected for full-text reading. Following the full-text review, 30 studies met the eligibility criteria and were included for further qualitative analysis. The study selection process is summarized in the flowchart in Appendix 3. A list of human clinical studies identified during screening but excluded from this review due to the preclinical inclusion criteria is provided in Appendix 5.

### Characteristics of included studies

All 30 preclinical in vivo studies included in this review utilized animal models to evaluate the bone regeneration capacity of chitosan and/or calcium carbonate (CaCO₃)-based scaffolds. The vast majority of studies employed small mammals due to their cost-effectiveness, well-understood physiology, and ease of handling. Rats were the most frequently used species (19 of 30 studies, or 63%), predominantly Sprague–Dawley and Wistar strains. Rabbits were used in 9 studies, mainly the New Zealand White strain. Other animal models included mice (2 studies), goats (1 study), beagle dogs (1 study), and rhesus monkeys (1 study). This diversity allowed for cross-species comparison of scaffold performance in both small and large defect environments. The calvarial bone was the most commonly targeted site, especially for critical-size defects, used in at least 8 studies, followed by defects in the mandible, femur, tibia, ulna, and sternum. Defect sizes varied widely depending on the anatomical site and species, ranging from 2 mm (tibial defects in rats) to 15 mm (ulnar defects in rabbits) and even larger in non-rodent models such as monkeys and goats. While gender, age, and weight were mentioned inconsistently, male animals were preferred in most rat studies to minimize hormonal variation. The healing or observation periods varied between 2 to 12 weeks, with 8 weeks being the most frequent endpoint for evaluating scaffold efficacy. In several studies, no details were provided on animal sex, age, or sample size, limiting the generalizability of the findings. Overall, the included studies represent a broad spectrum of animal models and bone defect types, providing a robust platform for assessing the osteogenic potential of chitosan and calcium carbonate-based scaffolds in vivo. A detailed summary of study subjects and defect models is presented in Appendix 4.

### Risk of bias assessment

The methodological quality of the included studies was assessed using SYRCLE’s Risk of Bias tool, which is specifically adapted for animal intervention studies. The tool encompasses ten domains, including selection bias, performance bias, detection bias, attrition bias, reporting bias, and other sources of bias. Out of the included studies, only a small proportion reported adequate random sequence generation (e.g., random assignment to groups), with the majority rated as “unclear” due to a lack of explicit methodological description. Similarly, allocation concealment and random housing were not mentioned in most studies, resulting in an “unclear” rating in these domains. Blinding procedures were rarely described. In most studies, the blinding of caregivers, investigators, and outcome assessors was not reported, resulting in a predominantly “unclear” risk of detection and performance bias. On the other hand, most studies reported their outcome data, with a low risk of attrition bias, as animal loss or exclusion was either absent or well accounted for. Likewise, selective outcome reporting was generally low, as the reported results aligned with the stated objectives and methods. Only a few studies were rated as having an overall “low risk of bias,” whereas the majority were categorised as “unclear,” indicating insufficient reporting rather than necessarily poor methodological quality. A complete summary of the SYRCLE assessment results is presented in Appendix 7.

### Outcome: chitosan and calcium carbonate scaffolds in bone regeneration

A total of 30 preclinical in vivo studies were included in this systematic review. These studies showcased a rich diversity, ranging from different animal models to various bone defect locations, scaffold compositions, and evaluation methods. The included articles were published between 2016 and 2025, with most studies conducted on rats or rabbits as experimental animals. Critical-size bone defects were created in various anatomical regions, such as the calvaria, mandible, femur, radius, and sternum, depending on the aim of each study. The meticulousness of the studies was evident in the range of defect sizes, from small (4 mm) to large (10 mm), and the healing observation periods, which varied between 2 and 12 weeks.

The experimental groups primarily involved scaffolds composed of chitosan (CS), calcium carbonate (CaCO₃), or their composites, sometimes combined with other biocompatible materials such as hydroxyapatite (HA), sodium alginate, or PLGA. While several studies focused on “pure” scaffold materials, others included biological enhancers such as growth factors, stem cells, or bioactive molecules. Control groups typically consisted of empty defects, autografts, or alternative biomaterial scaffolds (e.g., hydroxyapatite, collagen).

Outcomes were assessed using a robust combination of radiographic imaging (e.g., micro-CT, X-ray), histological examination, and, in some cases, biomechanical testing. This comprehensive approach was used to determine scaffold integration and bone regeneration capacity. Key outcome indicators included BV/TV, BMD, trabecular thickness, mineralisation, and new bone formation percentage. A complete summary of each study is presented in the data extraction table. Appendix 4.

## Discussion

### Conceptual framework: osteoconduction, osteoinduction, and defect filling

To understand the different outcome measures reported across the studies, it is crucial to apply a consistent mechanistic framework. In scaffold-based bone repair, three related yet distinct concepts help clarify (Arash and Hosseinpour 2020): osteoconduction—providing a porous, biocompatible matrix that facilitates host cell ingrowth and blood vessel formation (Li et al. 2021); osteoinduction—actively promoting progenitor cells to become osteoblasts via biological signals such as BMPs, growth factors, or introduced cells; and (Michael and Hagenmaier 2002) defect filling—a passive process that occupies space and may accelerate radiographic bridging without achieving true structural or functional recovery. This framework explains why measures like BV/TV, BMD, histological matrix formation, and biomechanical strength are not interchangeable: osteoconductive scaffolds can reliably increase BV/TV by providing a matrix but will not necessarily restore biomechanical function unless combined with osteoinductive signals or appropriate structural features.

To improve interpretative clarity and address heterogeneity across studies, this review adopts a stratified analytical framework that distinguishes between intrinsic scaffold effects and outcomes influenced by biological augmentation. This distinction is essential to avoid over-attribution of osteogenic outcomes to the scaffold material alone and to enable a more balanced and transparent comparison across studies. Based on this framework, the following sections interpret the findings through a comparative and mechanism-oriented perspective.

### Direct comparison: chitosan vs calcium carbonate (analytical synthesis)

Across the 30 included preclinical studies, chitosan-containing scaffolds were evaluated in 19 studies whereas CaCO₃-containing constructs appeared in 11 studies (some studies evaluated composites). Chitosan scaffolds reported new bone formation ranging from ~40% to 85%, whereas CaCO₃ scaffolds reported a narrower range (~50–70%) (see Results). These summary ranges suggest that chitosan systems achieved higher maximum reported bone formation (up to 85%) and a broader distribution of outcomes, likely reflecting the diversity of chitosan formulations and additive strategies (e.g., composites, crosslinkers). Mechanistically, chitosan contributes favorable cell-adhesive surfaces, intrinsic antimicrobial and immunomodulatory properties, and ease of chemical modification to tune degradation and porosity; CaCO₃ primarily offers mineral-like ionic exchange and pH-buffering that support mineral deposition and matrix maturation. Therefore, when comparing efficacy, chitosan appears to show greater variability but also a higher ceiling in reported osteogenic outcomes, while CaCO₃ appears to provide relatively consistent osteoconductive support—a difference that plausibly reflects their distinct modes of action. These comparative statements are presented cautiously because many high-performing results were obtained in the presence of biological enhancers (see next section).

### Cell-free versus bio-enhanced scaffold systems

A critical source of heterogeneity is whether scaffolds were tested as *cell-free/drug-free* constructs or as *bio-enhanced* platforms (growth factors, stem cells, exosomes, small molecules). Several included studies delivered potent osteoinductive agents (e.g., BMP-2, VEGF, ADSCs/hUSCs, or exosomes) and reported markedly higher new bone percentages. For example, Wang et al. reported approximately 86.6% new bone formation using a chitosan-based hydrogel delivering BMP-2 and VEGF with adipose-derived stem cells. Similarly, Liu et al. demonstrated improved mechanical strength in a large segmental ulnar defect repaired using a chitosan–biphasic calcium phosphate scaffold seeded with urine-derived stem cells. Conversely, multiple cell-free chitosan or CaCO₃ constructs also demonstrated intrinsic osteoconductive performance (e.g., Patlataya et al. (Patlataya et al. 2023) and Saveleva et al. (Saveleva et al. 2021) but generally with lower or more variable quantitative outcomes. Practically, this means the scaffold’s “apparent efficacy” must be deconvoluted into (a) intrinsic scaffold contributions, and (b) adjunct biological augmentation. For transparent interpretation we therefore recommend (and applied in our synthesis) to treat results from bio-enhanced systems as a separate analytic category and avoid attributing their gains solely to the base material. Therefore, direct comparisons between chitosan- and CaCO₃-based scaffolds should be interpreted with caution, as many of the highest reported outcomes are associated with adjunct biological interventions rather than the intrinsic properties of the scaffold materials alone.

### Subgroup interpretation: intrinsic vs augmented systems

To further address the lack of formal quantitative aggregation and improve analytical transparency, the included studies can be conceptually stratified into two subgroups (Arash and Hosseinpour 2020): intrinsic scaffold systems (cell-free or drug-free constructs), and (Li et al. 2021) bio-enhanced systems incorporating growth factors, stem cells, exosomes, or other bioactive agents. Across the included studies, bio-enhanced systems consistently demonstrated higher reported bone formation outcomes, frequently exceeding 70–85%, particularly in studies utilizing BMP-2, VEGF, or stem cell-based strategies. In contrast, intrinsic scaffold systems generally exhibited more moderate and variable outcomes, reflecting their dependence on host-driven regenerative processes. This pattern suggests that a substantial proportion of the observed regenerative efficacy in high-performing studies may be attributed to biological augmentation rather than scaffold composition alone. Therefore, direct comparisons between scaffold materials without accounting for adjunct biological interventions may lead to overestimation of intrinsic material performance. Although a formal meta-analysis was not feasible due to heterogeneity in study design, animal models, and outcome measures, this subgroup-based interpretation provides a more structured analytical approach and strengthens the validity of comparative conclusions presented in this review.

### Integrative biological model: immunomodulation, ionic buffering, and osteoblast differentiation

Beyond descriptive reporting of molecular markers, the included studies collectively suggest a coherent biological model linking material properties to osteogenic outcomes. Chitosan-based scaffolds exhibit notable immunomodulatory effects, as evidenced by reduced pro-inflammatory cytokines (e.g., IL-6 and TNF-α) and increased expression of osteogenic markers such as ALP, Runx2, and osteocalcin (OCN) in several studies. This anti-inflammatory microenvironment is critical during the early healing phase, as excessive inflammation can impair osteoblast differentiation and matrix mineralization.

In parallel, CaCO₃-containing scaffolds contribute through physicochemical mechanisms, particularly via Ca^2 +^ ion release and local pH buffering, which create a favourable microenvironment for mineral nucleation and osteoblast activity. The buffering effect may mitigate local acidosis in defect sites, thereby supporting cell viability and promoting osteogenic signaling pathways associated with matrix maturation and trabecular organization.

When interpreted together, these findings indicate that chitosan primarily enhances osteogenesis through immunomodulation and cell adhesion, while CaCO₃ supports mineralization through ionic exchange and microenvironmental stabilization. This complementary interaction provides a mechanistically plausible explanation for the improved bone regeneration observed in composite scaffolds, where reduced inflammatory signaling, enhanced osteogenic marker expression, and favourable ionic conditions converge to facilitate osteoblast differentiation and functional bone tissue formation. The mechanistic pathways underlying the osteogenic performance of chitosan and calcium carbonate scaffolds are summarized in Appendix 6, illustrating the biological and physicochemical processes that contribute to osteoblast differentiation and matrix mineralization.

### Translational relevance: defect size, load-bearing conditions, and functional recovery

Although several studies included large animal models such as goats and rhesus monkeys, the majority of the included experiments were conducted using small rodent calvarial defects (typically 4–8 mm), which are non-load-bearing and biologically more permissive to bone regeneration. Evidence from larger animal models further supports the translational potential of these scaffolds. For instance, Bangun et al. (Bangun et al. 2021) demonstrated enhanced bone formation in a goat alveolar cleft model using a hydroxyapatite/chitosan/gelatin scaffold combined with BMP-2 and mesenchymal stem cells, while Wang et al. (Wang et al. 2019) reported successful alveolar bone repair in rhesus monkeys using a BMP-2 gene-activated scaffold. In contrast, segmental long-bone defects (e.g., ulnar or radial defects up to 10–15 mm in rabbits) represent load-bearing conditions that more closely mimic clinical scenarios in oral and maxillofacial and orthopedic reconstruction. Importantly, high structural metrics such as BV/TV and BMD reported in calvarial models do not necessarily translate into functional mechanical recovery under physiological loading. For example, studies that incorporated biomechanical testing (e.g., three-point bending) demonstrated improved bending strength in scaffold-treated groups compared with untreated defects, yet mechanical properties remained inferior to native bone, indicating only partial functional restoration. This discrepancy highlights that structural bone formation (radiological and histological outcomes) and functional competence (mechanical strength and stiffness) should be interpreted separately. Therefore, while calvarial models are valuable for early osteoconductive assessment, load-bearing segmental defect models provide more clinically relevant evidence for translational application of chitosan and CaCO₃ scaffolds, particularly for maxillofacial reconstruction where functional load distribution is critical.

### Risk of bias and implications for effect-size interpretation

The SYRCLE assessment revealed frequent “unclear” ratings for sequence generation, allocation concealment, and blinding. This reporting gap introduces potential effect-size inflation, particularly in small rodent calvarial studies where high bone formation (e.g., >80%) was reported. In other words, when methodological safeguards are not reported, positive outcomes should be interpreted more cautiously. We therefore temper conclusions drawn from studies with unclear RoB and emphasize consistency across multiple, well-reported experiments as stronger evidence.

### Future perspectives: scaffold-mediated controlled delivery and small-molecule loading

In addition to structural and biological scaffold optimization, an emerging strategy in bone tissue engineering involves the use of scaffolds as controlled drug-delivery platforms. Chitosan- and CaCO₃-based scaffolds possess favorable physicochemical properties, including porosity, biodegradability, and ionic exchange capacity, which make them suitable carriers for osteoinductive small molecules and phytochemicals. One promising candidate is icariin, a bioactive flavonoid that has demonstrated significant osteogenic and anti-adipogenic effects through modulation of key signaling pathways. Mechanistic studies have shown that icariin suppresses the expression of adipogenic transcription factors (PPARγ, C/EBPα, and FABP4) and regulates the Notch signaling pathway, thereby promoting mesenchymal stem cell differentiation toward the osteoblastic lineage rather than adipogenesis.

Furthermore, icariin has been reported to inhibit N1ICD and Jagged1 expression while increasing Notch2 mRNA, suggesting a regulatory role in osteogenic signaling and bone metabolism. These molecular effects provide a biologically plausible rationale for incorporating icariin or similar osteoinductive phytochemicals into scaffold systems for sustained and localized delivery (Liu et al. 2017). Therefore, future scaffold designs may benefit from integrating drug-loading strategies that combine osteoconductive matrices (e.g., chitosan and CaCO₃) with controlled release of small osteogenic molecules, potentially enhancing both mechanistic efficacy and translational potential in maxillofacial bone regeneration.

## Limitations

Heterogeneity in scaffold fabrication, adjunct biology, animal species, defect models, and outcome metrics limit quantitative synthesis. Biomechanical endpoints were underreported and molecular mechanisms incompletely characterized in several studies. Finally, reporting deficits in methodological details increase uncertainty in effect estimates.

## Conclusion

Based on the currently available preclinical evidence, both chitosan- and CaCO₃-based scaffolds demonstrate promising potential for supporting bone regeneration in vivo. However, these findings should be interpreted with caution, as a substantial proportion of the reported outcomes are influenced by biological augmentation strategies rather than the intrinsic properties of the scaffolds alone.

Although radiographic and histological parameters such as BV/TV, BMD, and new bone formation consistently suggest regenerative activity, evidence for full functional biomechanical restoration remains limited. The current body of evidence indicates promising regenerative potential, although definitive conclusions remain limited by heterogeneity in study design and reporting quality. Further well-designed studies, particularly in large-animal, load-bearing models with improved methodological transparency are needed to strengthen confidence in the translational potential of these scaffold systems.

Together, these findings support the continued development of chitosan and CaCO₃-based scaffold systems as promising biomaterial platforms for bone tissue engineering, while emphasizing the importance of standardized experimental design to improve translational reliability. Future studies should prioritize standardized experimental designs and explicitly distinguish between intrinsic scaffold effects and adjunct biological contributions to enable more robust comparative evaluation.

## Data Availability

No datasets were generated or analysed during the current study.

## Appendix 1. Search strategies in different databases

**Table:**

Database	Search strategy
PubMed and Cochrane library	#1. rat OR rodent OR pig OR dog OR monkey OR rabbit OR mice OR goat OR murine OR animals OR animals[MeSH] #2. (chitosan OR poliglusam OR chitosan[MeSh]) (calcium carbonate OR CaCO3 OR limestone [MeSh]) AND (nano* OR “nanostructures” [MeSH]) AND Scaffold #3. (bone regenerate*) OR (bone format*) OR osteogen* OR (bone heal*) OR (bone repair*) OR (bone transplantation) OR (bone reconstruct*) OR ossificat* OR (bone augment*) OR (bone tissue engineering) [MeSH] OR “bone regeneration”[MeSH] #4. #1 AND #2 AND #3
WOS	#1. rat OR rodent OR pig OR dog OR monkey OR rabbit OR mice OR murine OR Goat OR animal* #2. chitosan OR poliglusam AND calcium carbonate OR CaCO3 AND scaffold #3. bone regenerate OR bone format OR osteogen OR bone heal OR bone repair OR “bone transplantation OR bone reconstruct*” OR ossificat OR bone augment OR bone tissue engineering #4. #1 AND #2 AND #3
Scopus	Scaffold OR scaffolds AND calcium AND carbonate OR vaterite AND chitosan OR cs AND bone AND tissue AND engineering AND regeneration

## Appendix 2. SYRCLE’s RoB tool for the evaluation of risk bias of studies.

**Table:**

Item	Type of bias	Domain	Review author judgement
1	Selection bias	Sequence generation	Was the allocation sequence adequately generated and applied?
2	Selection bias	Baseline characteristics	Were the groups similar at baseline, or were they adjusted for confounders in the analysis?
3	Selection bias	Allocation concealment	Was the allocation adequately concealed?
4	Performance bias	Random housing	Were the animals randomly housed during the experiment?
5	Performance bias	Blinding	Were the caregivers and/or investigators blinded from knowledge of which intervention each animal received during the experiment?
6	Detection bias	Random outcome assessment	Were animals selected at random for outcome assessment?
7	Detection bias	Blinding	Was the outcome assessor blinded?
8	Attrition bias	Incomplete outcome data	Were incomplete outcome data adequately addressed?
9	Reporting bias	Selective outcome reporting	Are reports of the study free of selective outcome reporting?
10	Other bias	Other source of bias	Was the study apparently free of other problems that could result in a high risk of bias?

## Appendix 4. Summary of included preclinical studies

**Table:**

No	Author & Year	Animal Model	Defect Model	Scaffold Type (I)	Comparator	Key Histological Findings	Biomechanical Test	Integration/Functional Outcome	Main Conclusion
**1**	Delan et al., 2022 (Delan et al. 2022)	New Zealand rabbits	unilateral maxillary bone defect	PVA nanofibers incorporating simvastatin/chitosan-TPP nanoparticles (SIM CS-TPP NPs)	Free SIM, PVA NFs with free SIM, unloaded PVA NFs, untreated control	Massive osteoblasts, new bone formation, no inflammation in G6 group	Not reported	High density, granulation tissue, neovascularization in defect site	SIM CS-TPP NPs-loaded PVA NFs showed superior bone regeneration and controlled release
**2**	Chen et al., 2020 (Chen et al. 2020)	New Zealand rabbits	bilateral femoral condyle lacunar bone defect (10 × 5 × 5 mm)	3D-printed HA/CMCS/PDA scaffold	Commercial hydroxyapatite-based scaffold (Bone3), blank control	HE and toluidine blue staining showed new bone, osteoblast activity; no inflammation	In vitro only: compressive strength = 2.0 ± 1.0 MPa, modulus = 8.9 ± 2.0 MPa; no in vivo data	Scaffold degraded and was replaced by new bone over 12 weeks	Excellent biodegradability and bone regeneration in vivo compared to commercial scaffold
**3**	Bohrnsen et al., 2021 (Böhrnsen et al. 2021)	Immunocompromised RNU rats	subcutaneous ectopic implantation (non-osseous)	CaCO₃ and HA scaffolds seeded with BMSC, HUVEC, or both	Non-seeded scaffolds (control)	↑ OP expression & mineralization in BMSC & BMSC+HUVEC, especially on CaCO₃	Not reported	↑ Vascularization (5.6 vessels/0.1 mm^2^ CaCO₃, 5.3 HA); BMSC highest in early angiogenesis	Cotransplantation improved osteogenesis & angiogenesis but no long-term superiority observed
**4**	Huang et al., 2020 (Huang et al. 2020)	Sprague-Dawley rats	bilateral critical-size calvarial defects (5 mm)	CaCO₃/MgO/CMC scaffold with BMP2	CaCO₃/MgO/CMC, CMC only, Negative Control (no scaffold)	Van Gieson, H&E, and Masson’s trichrome staining showed strong bone formation, collagen, and vessels	In vitro only: Young’s modulus = 7.27 kPa, Compressive strength = 3.02 kPa; no in vivo test reported	Near-complete defect closure, fusion with native bone, improved mineral density and structure	CaCO₃/MgO/CMC/BMP2 scaffold strongly enhanced bone regeneration via ERK1/2 and Akt signaling pathways
**5**	Roy et al., 2024 (Roy et al. 2024)	New Zealand white rabbits	Ti implant in femur and tibial heads	Titanium implants coated with Cissus quadrangularis-Chitosan Hydrogel (CqChH)	Commercially pure Ti implants (no coating)	Osteoid formation, active osteoblasts, vascularization (H&E, Masson Trichrome staining)	Yes – RTQ significantly higher at 12 weeks (75.96 vs 45.80 for Cp)	Enhanced osseointegration and implant stability in coated group	CqChH coating improved bone formation and implant stability; promising for early loading
**6**	Wang et al., 2020 (Wang et al. 2020)	Sprague-Dawley rats	bilateral critical-size calvarial defects (5 mm)	Thiolated chitosan/calcium carbonate (TCS-10%P24/CA) scaffold	TCS/CA, TCS-5%P24/CA, and control (no scaffold)	H&E and Masson’s trichrome showed new bone, osteoblasts, and vascularization in TCS-10%P24/CA	Not reported	Near-complete defect closure, high osteoblast activity, and angiogenesis in defect area	Scaffold promotes osteogenesis via sustained P24 release and BMSC differentiation support
**7**	Hendawy et al., 2022 (Hendawy et al. 2022)	New Zealand white rabbits	critical-size mandibular defect (10 mm)	Chitosan scaffold and Mineralized Plasmatic Matrix (MPM)	Saline (control group)	H&E and IHC showed osteoid formation, ↑ Collagen I, ↓ NF-κB & MMP9 expression	Not reported	Enhanced bone formation and better structural organization in MPM & chitosan groups	MPM and chitosan support osteogenesis and reduce inflammation in mandibular bone defects
**8**	Liao et al., 2024 (Liao et al. 2024)	Sprague–Dawley rats	critical-size femur defect (3 × 4 mm)	Injectable OD/CC/AS–Mg^2+^ hydrogel with oxidized dextran, chitosan, alendronate sodium, and Mg^2 +^	OD/CC hydrogel (no Mg^2 +^), control (no treatment)	Dense, layered bone, ↑COL-I, ↑OCN, ↓osteoclasts, ↑cartilage-like tissue (endochondral ossification)	Not reported	Improved vascularization (↑CD31), reduced inflammation (↓CD80), complete bone healing	Scaffold promotes osteogenesis, vascularization, anti-inflammation, and ossification; promising for bone regeneration
**9**	Wang et al., 2020 (Wang et al. 2020)	New Zealand rabbits	critical-size mandibular defect (8 mm)	Injectable hydrogel with chitosan, nHA, PLGA microspheres loaded with BMP-2/VEGF, seeded with ADSCs	Control, BMP-2 only, VEGF only	G4 (BMP-2+VEGF) had highest new bone % (86.56%), lamellar bone, and vascularization	Not reported	Scaffold fully integrated and resorbed in 12 weeks, CT value closest to native bone	Co-delivery of BMP-2/VEGF via injectable CS/nHA scaffold with ADSCs significantly enhances bone regeneration and vascularization
**10**	Penha et al., 2020 (Penha et al. 2020)	Wistar rats	tibial bone defect (2 × 2 mm)	Chitosan spheres with 5% and 20% D. ambrosioides extract	Chitosan only, and blood clot (no scaffold)	CA20 group showed significantly higher osteoblast and endosteal/periosteal formation across all timepoints	Not reported	CA20 group achieved complete histological bone repair at 30 days	Chitosan spheres with 20% extract promoted faster, superior bone regeneration with controlled release and low inflammation
**11**	Wang et al., 2025 (Wang et al. 2025)	Sprague–Dawley rats	bilateral 6 mm calvarial defect	GelMA/CMCS hydrogel with magnesium phosphate cement (GelMA-CM)	GelMA, GelMA-C, control (untreated)	Extensive new bone formation, high osteoblast activity, minimal fibrous tissue	Not reported	Scaffold retained shape at 8 weeks, vascularized bone, full coverage of defect	GelMA-CM scaffold enhanced osteogenesis via Mg^2 +^ release; promising for injectable bone repair
**12**	Deng et al., 2019 (Deng et al. 2019)	New Zealand rabbits	bilateral mandibular defect (13 × 6 × 4 mm)	3D-printed PLGA/nHA scaffold + chitosan/rhBMP-2 nano-sustained release system	PLGA/nHA only (blank control)	Dense trabecular bone, vascularization, higher osteocalcin and collagen I expression in treatment group	Not reported	45.5% new bone at 12 weeks vs. 19.2% in control; full integration, no inflammation, scaffold resorbed	Scaffold enhanced osteogenesis and vascularized bone formation; effective for mandibular bone regeneration
**13**	Da Cunha et al., 2023 (Da Cunha et al. 2023)	Wistar rats	5 mm circular defect in mandibular ramus	Anionic collagen/chitosan (Michael and Hagenmaier 2002) (Arash and Hosseinpour 2020): scaffold, with/without DPSCs	Clot only, DPSCs only	G4 (scaffold+DPSCs) had better interface, G2 (DPSCs only) had highest bone regeneration (51.17%)	Not reported	G2: 51.17%, G3: 31.31%, G4: 45.26% new bone after 6 weeks; scaffold partially integrated	Scaffold was osteoconductive and biocompatible, but DPSCs alone led to better bone formation in short term
**14**	Guo et al., 2019 (Guo et al. 2019)	New Zealand rabbits	6 mm calvarial defect	Chitosan-coated magnesium alloy (CS-Mg) membrane	Heal-All (commercial GBRM), blank control	CS-Mg and Heal-All showed significantly more new bone vs control; comparable bone regeneration between them	Not reported	No membrane residue at 12 weeks; CS-Mg promoted organized trabecular bone and angiogenesis	CS-Mg shows biocompatibility, controlled degradation, and bone regeneration comparable to commercial GBR membrane
**15**	Bangun et al., 2020 (Bangun et al. 2021)	Capra hircus (goat)	2 × 1.5 × 0.3 cm alveolar cleft defect	HA/Chitosan/Gelatin + BMP-2 + hUCMSCs	ICABG, HA/Chitosan/Gel + BMP-2	Most bone formed in HA/Chitosan/Gel + BMP-2 + hUCMSCs; minimal inflammation & angiogenesis at week 12	Not reported	Improved functional score, highest % new bone & density at week 12	Scaffold + BMP-2 + hUCMSCs enhanced bone regeneration & outperformed ICABG, with no donor site morbidity
**16**	Liu et al., 2021 (Liu et al. 2021)	New Zealand rabbits	15 mm critical-sized ulnar defect	Chitosan/Biphasic Calcium Phosphate (CS/BCP) scaffold + hUSCs	CS/BCP without cells, pure BCP scaffold, defect with no implant	Cell-CS/BCP group showed mature bone, osteocytes, collagen; superior to all other groups	Yes (3-point bending) - The bending strength of the cell-CS/BCP scaffold group (human urine-derived stem cells seeded on chitosan–biphasic calcium phosphate composite scaffolds) was significantly higher than that of the pure BCP scaffold group (*p* < 0.05). - No statistically significant difference in elastic modulus was observed among the experimental scaffold groups (CS/BCP vs. cell-CS/BCP), although both showed improved values compared to the untreated defect group (*p* < 0.05). - All treated groups exhibited lower mechanical performance compared to native ulna, confirming partial but not complete functional recovery at 12 weeks.	Highest bone volume and strength in Cell-CS/BCP group; scaffold integrated and degraded as expected	CS/BCP scaffold + hUSCs enhanced bone regeneration and mechanical properties; promising for large defects
**17**	Lohse et al., 2015 (Lohse et al. 2015)	132 Wistar rats	5 mm mandibular ramus critical-size defect	PDLLA/CaCO_3_ scaffold loaded with rhBMP2 and rhVEGF165 (various ratios)	Blank scaffold, rhBMP2 only, rhVEGF165 only, different BMP2/VEGF ratios	Ratio ~1 of VEGF/BMP2 enhanced both bone volume and density significantly	Not reported	Combined delivery promoted better remodeling, density, and early bone formation	VEGF + BMP2 (at ~ 1:1 ratio) enhances bone formation more effectively than BMP2 alone and allows dose reduction
**18**	Li et al., 2016 (Li et al. 2017)	SD rats (5 mm calvarial defect)	Beagle dogs (periodontitis model)	Thermosensitive CS/CSn hydrogel + pDNA-BMP2	CS/CSn hydrogel without pDNA-BMP2, empty defect	Increased trabecular bone, osteocytes, and mineralization in BMP2 group; control mostly fibrous tissue	Not reported	Sharpey’s fibers, new bone width & ALP highest in BMP2 group; no adverse tissue reaction	Scaffold enhanced alveolar bone regeneration, with good biocompatibility and injectable convenience
**19**	Hermenean et al., 2017 (Hermenean et al. 2017)	CD1 mice	5 mm critical-size calvarial defect	3D Chitosan/Graphene Oxide (CHT/GO) scaffold (0.5% & 3.0% wt)	Empty defect, CHT without GO	3.0 wt% GO enhanced bone formation, remodeling, ALP & osteogenic markers, scaffold integrated well without fibrous capsule	Not reported	Yes – robust bone formation, osteogenic marker upregulation, good scaffold-host integration	CHT/GO 3D scaffold (3 wt%) promotes osteogenesis & regeneration without growth factors or stem cells
**20**	Wang et al., 2019 (Wang et al. 2019)	Rhesus monkeys	10 × 10 × 5 mm critical alveolar defect	3D-printed bioglass + BMP/CS + rBMSCs	Sham-op, 3D-BG only, 3D-BG + BMP/CS	Mature trabecular bone, cortical continuity, marrow formation, no inflammation	Not reported	Full scaffold resorption, host-bone integration, organized structure and vascularization	Significantly enhanced bone regeneration in primates, promising for alveolar bone repair
**21**	Jahan et al., 2020 (Jahan et al. 2020)	C57BL/6 mice	rod-fixed tibial fracture	Injectable GDP-crosslinked Chitosan-Hydroxyapatite + Pyrophosphatase (CS75HAP)	CS only, CS75HA, SHAM	CS75HAP: highest osteoid, ALP, mineralization; trabecular bone observed	Not reported	Yes – strong integration, bone remodeling, callus formation, no adverse reaction	CS75HAP promotes osteogenesis and mineralization in vivo, better than other scaffold types
**22**	Saveleva et al., 2021 (Saveleva et al. 2021)	White male rats	non-critical femoral defect	PCL scaffold mineralized with vaterite (CaCO3), with/without ALP	Unmineralized PCL, PCL + LPS (neg. control), simulated defect	PCL/CaCO_3_/ALP: 62.9% new bone, strong osseointegration, mature trabeculae, angiogenesis	Yes – tensile test (Universal Testing Machine, 20 mm/min); Young’s modulus: 3 ± 0.5 MPa (mineralized), 6.5 ± 1.5 MPa (non-mineralized)	Yes – complete defect repair in 75% cases (PCL/CaCO_3_/ALP group), scaffold closely adhered to defect edges	Vaterite-mineralized PCL scaffolds (especially with ALP) significantly promote osteogenesis, vascularization, and regeneration with sufficient mechanical support.
**23**	Man et al., 2016 (Man et al. 2016)	New Zealand white rabbits (2.5–3.0 kg)	full-thickness cartilage defect (4 mm × 1 mm)	Chitosan hydrogel–demineralized bone matrix (CS/DBM) hybrid scaffold with allogenic chondrocytes	CS-only, DBM-only, and microfracture (MF) groups	CS/DBM: smooth surface, high COL2, low COLX expression, best histological score	Yes – nanoindentation: Elastic modulus = 1.25 ± 0.18 MPa, Hardness = 0.12 ± 0.01 MPa	Yes – tissue in CS/DBM group integrated smoothly with surrounding cartilage, indistinct boundary at 24 weeks	CS/DBM with allogenic chondrocytes is effective for one-step cartilage repair with strong mechanical and histological outcomes
**24**	Oryan et al., 2017 (Oryan et al. 2017)	Sprague-Dawley rats	bilateral 5-mm critical-sized radial defect	Chitosan (CS), Platelet Gel (PG), CS-PG combination	Untreated defect, autograft from contralateral radius	PG: high bone formation & integration; CS: poor healing & inflammation; CS-PG: improved CS performance but reduced PG efficacy	Autograft: Max load 29.8 ± 4.1 N, Strain 1.53 ± 0.23%, Stress 11.1 ± 1.6 MPa, Stiffness 78.9 ± 5.9 N/mm PG: 28.7 ± 3.7 N, 1.67 ± 0.21%, 10.3 ± 1.4 MPa, 72.3 ± 6.4 N/mm CS-PG: 19.9 ± 2.4 N, 2.41 ± 0.29%, 7.5 ± 1.1 MPa, 58.6 ± 5.2 N/mm CS: 10.1 ± 1.8 N, 3.12 ± 0.31%, 3.6 ± 0.7 MPa, 35.4 ± 4.3 N/mm Defect: 9.5 ± 2.0 N, 3.28 ± 0.35%, 3.2 ± 0.6 MPa, 33.7 ± 3.8 N/mm	Yes – PG showed nearly complete bridging; CS-PG moderate; CS poor healing	PG enhanced bone regeneration and mechanics, comparable to autograft. CS alone ineffective. CS-PG helped CS but reduced PG’s full effect.
**25**	Makar et al., 2023 (Makar et al. 2023)	Sprague–Dawley rats (*n* = 15)	6 mm calvarial critical-size defect	GA/Chitosan/nHA cross-linked with Genipin or FeCl₃	Empty defect (negative control)	Genipin group showed higher osteoid bone formation and cellular infiltration than FeCl₃; control had granulomatous tissue	Yes – Compressive strength test: Genipin: 21 ± 1.57 MPa; FeCl₃: 13.7 ± 2.1 MPa. Genipin enhanced strength by ~61%.	Yes – Genipin improved porosity, integration, and hydrophilicity vs. FeCl₃; more mature bone tissue observed in Genipin group.	Genipin cross-linker significantly enhanced mechanical, structural, and regenerative properties vs. FeCl₃; potential for bone scaffolds.
**26**	Wang et al., 2024 (Wang et al. 2024)	New Zealand rabbits (*n* = 24)	8 mm mandibular bone defect	nHA/CS/PLGA scaffold loaded with ADSC-derived exosomes and BMSCs	Control (no scaffold), nHA/CS/PLGA only, nHA/CS/PLGA + EXO	At 12 weeks: control = persistent defect; Graft-EXO + BMSCs = nearly complete regeneration with lamellar bone formation	Not reported	Yes – better scaffold degradation, integration, and mature bone formation in Graft-EXO + BMSCs group	Scaffold + EXO + BMSCs significantly improved mandibular bone regeneration; promising for maxillofacial repair applications.
**27**	Moser et al., 2017 (Moser et al. 2017)	Wistar rats (*n* = 24)	bilateral mandibular defect and ectopic site (gluteal muscle)	PDLLA/CaCO₃ scaffold loaded with rhBMP2 (24, 48, 96 µg), retarded vs. rapid release	Empty defect, blank scaffold, rapid vs. retarded delivery of BMP2	Retarded release induced higher bone volume in ectopic sites at low & medium doses; in mandibular sites, both release modes induced comparable bone formation; AP & Runx2 expression correlated with bone in retarded but not rapid release groups	Not reported	Yes – especially in ectopic sites; better coordination of osteogenic markers and bone formation with retarded delivery	Retarded delivery of rhBMP2 more effective in low osteogenic environments (ectopic); may not add benefit in mandibular defects with high innate regenerative capacity. Supports reduced dosage with controlled delivery in clinical applications.
**28**	Wang et al., 2016 (Wang et al. 2016)	Sprague-Dawley rats (*n* = 12)	10 mm critical-size calvarial defect	PLGA microspheres containing amorphous CaCO_3_ (polyP) and/or ACC	β-TCP, polyP-only	PolyP/ACC microspheres showed higher tissue infiltration, mineralization, and ossification centers than controls. Combined scaffold induced higher gene expression (CA IX and ALP).	Yes – nanoindentation showed 2.38 MPa vs. 0.63 MPa (β-TCP), and 1.74 MPa (polyP)	Yes – polyP/ACC microspheres nearly restored defect after 12 weeks, with dense mineralized and connective tissue	Combined amorphous polyP and ACC in PLGA microspheres significantly enhanced bone regeneration and mineralization compared to controls and individual components. Promising for regenerative bone implants.
**29**	Patlataya et al., 2023 (Patlataya et al. 2023)	Female Wistar rats (*n* = 84)	4 × 5 × 5 mm^3^ mandibular critical-sized defect (angle of mandible)	Lyophilized gel of CH–SA–HA (chitosan–sodium alginate–hydroxyapatite)	Blood clot only (natural healing)	CH–SA–HA group showed early signs of bone formation as early as day 7. At 2 weeks, bone volume significantly increased. Full closure occurred at 8 weeks with high mineralization. Control group showed delayed osteoid and osteoblast appearance.	Not reported	Yes − 100% closure of bone defect at 8 weeks in scaffold group with organized osteons and Haversian systems	CH–SA–HA scaffold accelerated early bone healing and provided complete regeneration of critical-sized mandibular defect in rats. Promising scaffold for maxillofacial reconstruction.
**30**	Salehiamin et al., 2022 (Salehiamin et al. 2022)	Wistar rats (*n* = 60)	sternotomy model (median split of sternum, ~12 mm incision)	Chitosan scaffold with Periostin (CT-Post), and combinations with OCP	Bone wax (BW), untreated, CT, CT-OCP, CT-OCP-Post	CT-Post group showed complete scaffold degradation, highest osteocyte number, trabecular bone volume (TBV), and lowest fibrous connective tissue (FCT). BW group showed poor healing and retained material. CT-Post also elevated Runx2, OPG, RANKL expression and ALP activity while decreasing IL-6 and TNF-α at day 7 and 21.	Not reported	Yes – CT-Post provided full integration at defect site with high bone mass and low inflammatory cytokine levels	Chitosan-Periostin scaffold significantly enhanced bone healing, suppressed inflammation, and promoted osteogenesis in sternum defect model. OCP addition reduced efficacy, suggesting interaction with Post release. CT-Post may serve as a potential alternative to bone wax for post-sternotomy regeneration.

## Appendix 5. Human studies excluded after full-text screening

**Table:**

No	Author	Year	Study Type	Reason for Exclusion	Relevance to Review	DOI
1	Meenakshi & Sankari	2021	RCT	Human clinical study - patients with chronic periodontitis	Evaluated chitosan nanohydrogel for bone regeneration in intrabony defects; provides clinical efficacy data	10.1177/2320206821998574
2	Kudoh et al.	2019	Clinical Trial	Human clinical study - maxillary sinus floor augmentation	First-in-human trial of carbonate apatite granules; demonstrates safety and efficacy in humans	10.1016/j.joms.2018.11.026
3	Kowalczyk et al.	2021	Clinical Study	Human clinical study - guided bone regeneration	Evaluated chitosan-human bone composite granulates; relevant for GBR applications	10.3390/ijms22052324
4	Cicciu et al.	2019	Systematic Review	Human clinical studies review - dentistry applications	Reviewed chitosan use in dentistry; provides overview of clinical evidence	10.3390/md17070417
5	Kattimani et al.	2019	RCT	Human clinical study - bone graft substitute evaluation	Evaluated eggshell-derived nano-hydroxyapatite as bone graft; clinical comparative study	10.1177/0885328219863311
6	Eshwar et al.	2023	RCT	Human clinical study - periodontal intra-bony defects	Evaluated fucoidan-containing chitosan hydrogel; clinical efficacy in periodontal regeneration	10.3390/gels9070573
7	Resende et al.	2019	RCT	Human clinical study - alveolar bone repair	Evaluated nanostructured carbonated hydroxyapatite; clinical trial for dental bone repair	10.3390/ma12223645
8	Fukuba et al.	2023	Pilot Study	Human clinical study - periodontal regeneration	Evaluated carbonate apatite granules for intrabony defects; prospective pilot study	http://10.1016/j.reth.2023.08.002
9	Logoluso et al.	2016	Pilot Study	Human clinical study - implant coating safety	Evaluated calcium-based antibiotic-loaded bone substitute; pilot clinical safety study	10.7150/jbji.17586
10	Rahyussalim et al.	2019	Clinical Study	Human clinical study - bone substitute material	Evaluated carbonate apatite as bone substitute; clinical application review	10.13181/mji.v28i1.2681
11	Nunn et al.	2024	Systematic Review	Human clinical studies review - carbonate apatite	Systematic review of carbonate apatite granules; clinical evidence synthesis	10.3389/fdmed.2024.1418039
12	Gerova-Vatsova et al.	2025	Systematic Review	Human clinical studies review - chitosan materials	Evaluated effectiveness of chitosan-modified bone regeneration materials	10.3390/pharmaceutics17050665
13	Hafez et al.	2021	Clinical Study	Human clinical study - jaw bone defects	Evaluated chitosan/hydroxyapatite scaffold on osseous defects in jaw bones	10.21608/ADJALEXU.2021.144906
